# Effective mechanisms of water purification for nitrogen-modified attapulgite, volcanic rock, and combined exogenous microorganisms

**DOI:** 10.3389/fmicb.2022.944366

**Published:** 2022-08-10

**Authors:** Yao Zheng, Yuqin Wang, Xiaoxi Yang, Jiancao Gao, Gangchun Xu, Julin Yuan

**Affiliations:** ^1^Key Laboratory of Integrated Rice-Fish Farming Ecology, Ministry of Agriculture and Rural Affairs, Freshwater Fisheries Research Center (FFRC), Chinese Academy of Fishery Sciences (CAFS), Wuxi, China; ^2^Wuxi Fishery College, Nanjing Agricultural University, Wuxi, China; ^3^Key Laboratory of Healthy Freshwater Aquaculture, Ministry of Agriculture and Rural Affairs, Key Laboratory of Fish Health and Nutrition of Zhejiang Province, Zhejiang Institute of Freshwater Fisheries, Huzhou, China

**Keywords:** purification materials, exogenous microorganisms, water purification enhancement, microbial enzyme activity, metagenome

## Abstract

The study tested the water purification mechanism of the combination of microorganisms and purification materials *via* characteristic, enzymatic, and metagenomics methods. At 48 h, the removal rates of total nitrogen, total phosphorous, and Mn chemical oxygen demand in the combination group were 46.91, 50.93, and 65.08%, respectively. The alkaline phosphatase (AKP) activity increased during all times tested in the volcanic rock, Al@TCAP, and exogenous microorganism groups, while the organophosphorus hydrolase (OPH), dehydrogenase (DHO), and microbial nitrite reductase (NAR) activities increased at 36-48, 6-24, and 36-48 h, respectively. However, the tested activities only increased in the combination groups at 48 h. Al@TCAP exhibits a weak microbial loading capacity, and the Al@TCAP removal is primarily attributed to adsorption. The volcanic rock has a sufficient ability to load microorganisms, and the organisms primarily perform the removal for improved water quality. The predominant genera *Pirellulaceae* and *Polynucleobacter* served as the sensitive biomarkers for the treatment at 24, 36-48 h. Al@TCAP increased the expression of Planctomycetes and Actinobacteria, while volcanic rock increased and decreased the expression of Planctomycetes and Proteobacteria. The growth of Planctomycetes and the denitrification reaction were promoted by Al@TCAP and the exogenous microorganisms. The purification material addition group decreased the expression of Hyaloraphidium, Chytridiomycetes (especially Hyaloraphidium), and Monoblepharidomycetes and increased at 36-48 h, respectively. Ascomycota, Basidiomycota, and Kickxellomycota increased in group E, which enhanced the nitrogen cycle through microbial enzyme activities, and the growth of the genus *Aspergillus* enhanced the phosphorous purification effect.

## Introduction

In intensive aquaculture systems, such as cage and pond cultures, extra nutrients are often discharged without treatment (Santos et al., 2016). Approximately, 75% of aquaculture feed residues are converted into nitrogen and phosphorus in wastewater (Serpa et al., 2013). The use of purification materials can effectively reduce the contents of nutrients, such as nitrogen and phosphorus. Research on the actual treatment effect of aluminum-loaded attapulgite (Al@TCAP) on black and odorous water found that Al@TCAP can effectively reduce ammonia nitrogen (NH_3_-N) and total phosphorous (TP) in water (Yin et al., 2019). Volcanic rock can be used in the remediation of heavy metals (de Anda et al., 2019) and wastewater treatment (Wan et al., 2020).

However, the more effective and higher pollutant removal rate requires longer hydraulic resident times (HRT) in the tune of weeks to months. First, these nutrients typically promote the growth of aquatic plants because they readily absorb and utilize certain dissolved inorganic substances, such as ammonia, urea, and phosphate (i.e., PO_4_^3–^-P, Franchino et al., 2016). Through the calculation, with the combined ammonia removal efficiencies of several nitrifying bacteria, the removal rate of NH_3_-N reached 71% (Sangnoi et al., 2017). The microorganisms have been used to convert dissolved organic matter into harmless substances, and the commercial micro-ecological products include effective microorganisms (EM), Bacillus, Streptococcus, Lactobacillus, and photosynthetic bacteria (Kuebutornye et al., 2019). The exogenous microorganisms were used to enhance the water purification effect when combined with purification materials that can provide a microorganism-loaded environment (Ren et al., 2021; Yin et al., 2022). The nitrifying and phosphorus-accumulating bacteria were enriched when added microorganisms in the activated sludge (Kuśnierz et al., 2022).

In Jiangsu province of China, the wastewater treatment system named “two dams and three districts” was constructed for water purification to remove suspended solids, nitrogen, and phosphorus. Two major problems in wastewater treatment systems should be solved urgently: the mechanism of the crosstalk in these interactive biochemical reactions (containing organics degradation, nitrification, denitrification, and microbial activities), and the enhancer method of water purification within the shorter HRTs in actual sewage treatment systems, even 40% removal rate obtained in our previous study *via* “Al@TCAP-volcanic rock + bacteria preparation + activated sludge.” Our recent study showed that the enhanced removal effect in this wastewater treatment system occurred when adding microorganisms and activated sludge (Yin et al., 2022), while the mode of action has not been determined.

In this study, the key factors governing the technical feasibility of the wastewater treatment system are evaluated: (1) the enhanced effect of exogenous microorganisms based on the two purification materials (Al@TCAP and volcanic rock) in the wastewater treatment system (superficial characteristic, nutrient removal, and enzyme activity), (2) the useful enhancers of bacterial and fungal category *via* 16S rRNA- and ITS-Seq methods. The purpose of this experiment was to explore the water purification mechanism effectiveness of a combination of microorganisms and purification materials.

## Materials and methods

### Experimental design and sampling

The experimental wastewater was rearing water for tilapia *Oreochromis niloticus* from the base center of FFRC-CAFS. The water quality of the wastewater was determined during the experiment. The selected purification materials, Al@TCAP, and volcanic rock were prepared in our laboratory using the method reported by Yin et al. (2019) and Wan et al. (2020). The Al@TCAP and volcanic rock distribution method was tile and accumulation based on the optimal purification effect in the pre-experiment, and the total added amount was 6 g/L. The composed purification material added half of the amount (6 g/L) of Al@TCAP and volcanic rock. The exogenous microorganisms, included *Bacillus licheniformis*, *Bacillus sp.* (Jiangsu Suwei microbial research Co., Ltd.) (Ren et al., 2021), and activated sludge (Sk Hynix Semiconductor (Wuxi) Co., Ltd.) (Santorio et al., 2021; Kuśnierz et al., 2022), were named as three bacteria liquids. Their concentrations were 1 × 10^8^-1 × 10^9^ CFU/ml of a bacterial liquid dosage of 1 mg/L following Murray et al. (1981).

Five groups were used in the study: A, the control group (without purification materials or bacteria liquid); B, 6 g/L volcanic rock; C, 6 g/L Al@TCAP; D, 1 mg/L three bacteria liquids (actual detected as 4.6 × 10^8^ CFU/mL); and E, 6 g/L Al@TCAP + 6 g/L volcanic rock + 1 mg/L three bacteria liquids (4.6 × 10^8^ CFU/mL). First, the water samples collected at 0 h (s1), 6 h (s2), 12 h (s3), 24 h (s4), 36 h (s5), and 48 h (s6) after the start of the experiment were used to determine water quality and enzyme activity. The reasons for the sampling period selection mainly included the active enzymatic (Otte et al., 1996) and retention time (Alloul et al., 2021) for function, and the short centralized drainage time duration in the field culture of Jiangsu province. The purification materials were loaded with or without exogenous microorganisms, and their superficial characteristics were identified using SEM. The samples were fixed with 2.5% glutaric acid fixative solution before the electron microscopy analysis and then dried at 105°C for 6 h before the full-pore adsorption and desorption experiments.

### Superficial characteristics of the purification material

The SEM method for the purification material is described in our previous studies (Zheng et al., 2020). The sample to be tested was vacuum degassed at 250°C for 3 h, the adsorption substance was N_2_, and the adsorption temperature was 77.3 K. The static adsorption equilibrium volume method was used to determine the gas adsorption isotherm of the sample. A specific surface area and porosity analyzer, ASAP 2460, was used for the full-pore adsorption and desorption experiments. Finally, the measured adsorption isotherm data were collected. The data for adsorption and desorption cumulative surface area of pores of the samples were determined.

### Water quality determination

The total nitrogen (TN), NH_3_-N, NO_3_^–^-N, nitrite nitrogen (NO_2_^–^-N), TP, PO_4_^3–^-P, and Mn chemical oxygen demand (COD_Mn_) were analyzed as described (State EPA of China, 2002). The dissolved oxygen (DO) was measured *in situ* with a YSI EXO2 multiparameter sonde (United States). The water removal rate was determined by dividing the decrease in water quality (treatment group minus control) by the original value.

### Enzyme activity

A series of enzyme-linked immunosorbent assay kits including those for microbial ammonia monooxygenase (AMO), nitrate reductase (NAR), nitrite reductase (NIR), alkaline phosphatase (AKP), organophosphorus hydrolase (OPH), and dehydrogenase (DHO) were purchased from Meimian Biotechnology Company. The activities of AMO, NAR, NIR, AKP, OPH, and DHO were tested in the samples according to the protocols. Simply, the samples and embedded monoclonal antibodies were added to the microwells and then combined with a horseradish peroxidase enzyme. After thoroughly washing the samples, the substrate tetramethylbenzidine was revealed first in blue and then yellow before the acid stop solution was added. The intensity of the color was positively correlated with the enzyme tested in the sample. The absorbance (OD value) was measured at 450 nm using a microplate reader (Biomarker Technologies Corporation, Beijing, China). The activity concentration of the enzyme in the sample was calculated using the standard curve.

### Metagenome

The metagenome used both 16SrRNA (bacterium) and ITS (fungus) sequences. In the 16SrRNA-Seq, the samples for M01-M03, M04-M06, M07-M09, M10-M12, and M13-M15 were named as groups A, B, C, D, and E at 0 h, respectively. The other samples for M16-M90 corresponded to the A-E group at 6, 12, 24, 36, and 48 h orderly. Total bacterial DNA extraction from samples, polymerase chain reaction amplification, and the methods described in Zheng et al. (2018) were used in the study, while for fungi, we followed the methods described in Fan et al.’s (2018) study. All PCR products were quantified using the Quant-iT dsDNA HS Reagent and pooled. High-throughput sequencing analysis of the fungi and bacterial rRNA genes was performed on the purified, pooled sample using the Illumina HiSeq 2500 platform (2 × 250 paired ends) at Biomarker Technologies Corporation (Beijing, China). The original data were spliced (FLASH, version 1.2.11), and the spliced sequences were quality filtered (Trimmomatic, version 0.33), and the illusions (UCHIME, version 8.1) were removed to obtain high-quality tag sequences. The sequences clustered with more than 97% similarity (USEARCH, version 10.0), while the operating taxonomical units (OTUs) were filtered with 0.005% as the threshold. Silva (release 132) was selected for the bacterial 16S database, and Unite (release 8.0) was chosen for the fungal ITS database. Using the RDP Classifier for species annotation, the confidence threshold was 0.8 (version 2.2). For multiple comparisons, the bacteria were PyNAST (version 1.2.2), the fungus was ClustalW2, and the phylogenetic tree was established using the neighbor-joining method.

The research aimed to identify the core microbiome in the same group and the presence of core bacteria that affect nitrification and denitrification in all the samples. The community indices we applied here include Chao1, Ace, Shannon, and Simpson indexes. A 0.5%, relative abundance threshold was used, which focused our analysis on PCR reproducible OTUs. To find the quality of 16SrRNA-Seq and ITS-Seq affected by the time (from M01 to M90) and different treatments (from groups A-E), the average data of five groups were taken as one group to obtain the new data for 0, 6, 12, 24, 36, and 48 h. The variation was calculated by selecting a significant increase or decrease in the bacterial community in a database constructed through a series of comparisons (e.g., s1 *vs.* s2 or s1 *vs.* s3). Differences can vary from the phylum to species level.

### Data analysis

Mean comparison was performed using Fisher’s least significant difference test and the Duncan multiple range test with a significance level of *p* < 0.05, and the relevant graphs were drawn in Origin 9.4.

## Results

### Superficial characteristics

**FIGURE 1:**
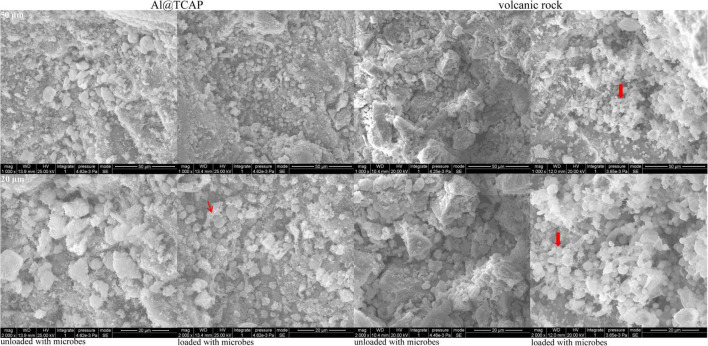
SEM results for Al@TCAP and volcanic rock when loaded or unloaded with microbes. The red arrow showed loaded with microbes and bacterial film formation. The Al@TCAP surface was rough, uneven, and exhibited many granular protrusions. The surface of the granular protrusions was groove shaped, and there were pores under the granular protrusions. The surface of the volcanic rock had granular protrusions and more pores. The protrusion surface was rough and exhibited a block shape.

There were fewer microorganisms on the surface of the Al@TCAP, and no bacterial film was formed (Figure 1). The volcanic rocks carrying microorganisms contained many organisms that formed a bacterial film. The adsorption and desorption cumulative surface area of pores in Al@TCAP were 28.17 and 38.30 m^2^/g, respectively, while for volcanic rock, it became 9.59 and 18.22, respectively. The surface area indices of the Al@TCAP were higher than those of the volcanic rock.

### Water quality

**FIGURE 2:**
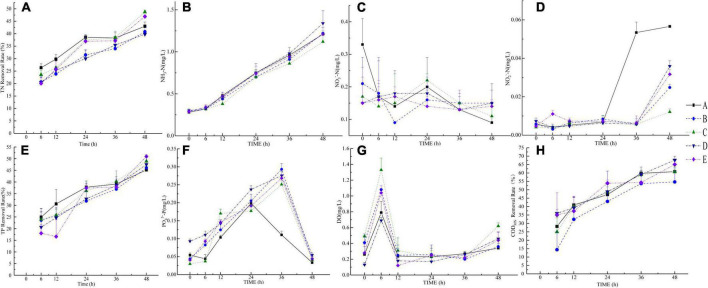
Measurement results of eight water quality indicators. **(A–H)** pictures are TN (removal rate), NH_3_-N, NO_3_^–^-N, NO_2_^–^-N, TP (removal rate), PO_4_^3–^-P, DO, and COD_Mn_ (removal rate), respectively.

In the nitrogen cycle, the TN concentrations in groups C and E were lower at 48 h than that of the other groups (Figure 2A). The TN removal rate of group A was higher than that of other groups at 6–36 h and lower than that of groups C and E at 48 h. The NH_3_-N in each group displayed an upward trend. The concentration of NH_3_-N in group C was lower than that in other groups after 12 h, and the frequency of NH_3_-N in group D was higher than that in the other groups after 24 h (Figure 2B). The NO_3_^–^-N concentration in group A was higher than that in the other groups at 0 h, the NO_3_^–^-N concentration in group B was higher than that in group A at 6 h, and the NO_3_^–^-N concentrations were higher in groups C, D, and E at 12 h. The frequency of NO_3_^–^-N in group C was higher than that in group A at 24 h. The concentrations of NO_3_^–^-N in groups C and B were higher than that in group A at 36 h, and the concentrations of NO_3_^–^-N in the other groups were higher than that in group A at 48 h (Figure 2C). The concentration of NO_2_^–^-N in group A was low between 0 and 24 h. The concentration of NO_2_^–^-N in group A increased during 24-36 h, and the concentration of NO_2_^–^-N was higher at 36 and 48 h than that in the other groups. The NO_2_^–^-N concentrations in groups B, C, D, and E were low between 0 and 36 h, and they increased at 36-48 h (Figure 2D).

The TP removal rate was the highest at 6-24 h. The TP removal rate in group C was higher than that in group A at 36 h. The removal rates of TP in groups C and E were higher than that in group A at 48 h. The PO_4_^3–^-P concentration typically exhibited an upward-decreasing trend (Figure 2E). Group A starts to decrease after a peak at 24 h, and the other groups drop after reaching a peak at 36 h. The PO_4_^3–^-P concentration in group D was higher than that in group A at 0 h, and the PO_4_^3–^-P concentrations in groups B, D, and E were higher than that in group A at 6 h. The PO_4_^3–^-P concentrations in groups B and D were higher than that in group A at 12 h (Figure 2F). The DO concentration in group D was lower than that in other groups at 0, 6, and 24 h, and the DO concentration in group C was higher than that in other groups at 0, 6, and 48 h. At 48 h, the DO concentration in group A was lower than that in the other groups. The DO concentration of each group increased at 0-6 h, decreased at 6-12 h, and then slowly increased at 12-48 h (Figure 2G). The COD_Mn_ removal rates in groups D and E were higher than that in group A at 6 h, and the COD_Mn_ removal rate in group A was higher than that in other groups at 12 and 36 h. The COD_Mn_ removal rates were higher in groups C, D, and E than in group A at 24 and 48 h. The COD_Mn_ removal rates in groups D and E were higher than that in group A (Figure 2H). At 48 h, the removal rates of total nitrogen, total phosphorous, and Mn chemical oxygen demand in the purified materials plus the three exogenous microorganisms were 46.91, 50.93, and 65.08%, respectively.

### Enzyme activities

**FIGURE 3:**
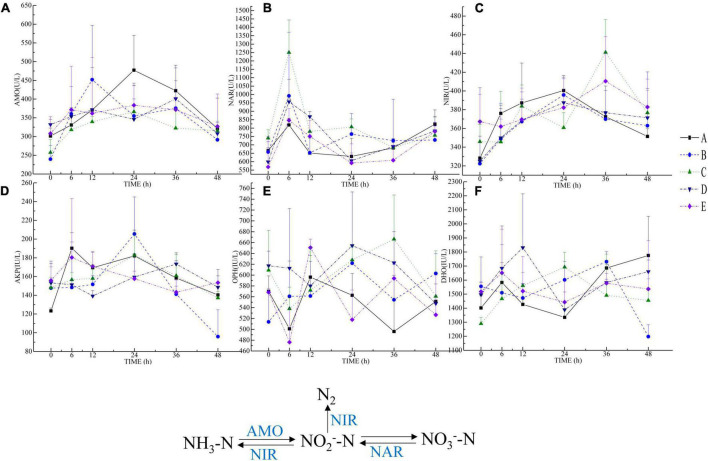
Results of microbial enzyme activity determination. **(A–F)** pictures are AMO, NAR, NIR, AKP, OPH, and DHO, respectively. The NIR, NAR, and AMO participate in the nitrogen cycle. NIR degrades NO_2_^–^-N to N_2_ or NH_3_-N, reducing the accumulation of NO_2_^–^-N in the environment. NAR is an inducible enzyme that can directly reduce NO_3_^–^-N to NO_2_^–^-N. AMO catalyzes ammonia oxidation by helping ammonia-oxidizing bacteria, which can help ammonia-oxidizing bacteria convert NH_3_-N to NO_2_^–^-N. AKP can catalyze the decomposition of organic phosphorus and release orthophosphate, while OPH can decompose organic phosphorus compounds in water and reduce organic phosphorus compound pollution. DHO is an oxidoreductase that participates in the decomposition of organic matter.

In the nitrogen cycle, the ammonia monooxygenase (AMO) activity was higher in groups D and E than in group A at 0 h, in groups B, D, and E at 6 h than in group A, in group B at 12 h than in group A, and in group A at 24 and 36 h. Compared with other groups, group E exhibited more activity than group A at 48 h (Figure 3A). The nitrate reductase (NAR) activity was higher in group C than in group A at 0 h, and it was higher in each group that group A at 6 and 12 h. Groups B and C exhibited more NAR activity than group A at 24 h, and group B was more active than group A at 36 h. Group A exhibited more activity than the other groups at 48 h (Figure 3B). The nitrite reductase (NIR) activity was higher in groups C and E than in group A at 0 h, and group A activity was higher than that in other groups at 6-24 h, groups C, D, and E were more active than group A at 36 h, and all other groups exhibited more activity than group A at 48 h (Figure 3C).

It can be seen that the alkaline phosphatase (AKP) activity of group A was lower than that of the other groups at 0 h and higher than that of other groups at 6 h (Figure 3D). AKP activity was higher in group E than in group A at 12 h, groups B and C were more active than group A at 24 h, groups C and D were more active than group A at 36 h, and groups D and E were more active than group A at 48 h. The organophosphorus hydrolase (OPH) activity was higher in groups C and D than in group A at 0 h, groups B, C, and D exhibited greater OHP activity than group A at 6 h, group E activity was higher than group A at 12 h, and groups B, C, and D were more active than group A. Each group exhibited more OPH activity than group A at 36 h, and groups B, C, and D were more active than group A at 48 h (Figure 3E).

The dehydrogenase (DHO) activity was higher in groups B, D, and E than in group A at 0 h, groups D and E exhibited more DHO activity than group A at 6 h, and all other groups exhibited higher activity than group A at 12 and 24 h, and group B exhibited higher activity at 36 h. Group A was more active than other groups (except group B) at 36 and 48 h, and group activity A was higher than that of the other groups at 48 h (Figure 3F).

### α/β-diversity for operating taxonomical units *via* 16SrRNA-Seq method

The 16S rRNA test results showed that Planctomycetes, Proteobacteria, and Bacteroidetes are the three primary group phyla. The predominant genera are *Pirellulaceae*, *Polynucleobacter*, and other Patescibacteria, Planctomycetes, Actinobacteria, Dependentiae, and Deinococcus-Thermus displayed significant differences between groups (Supplementary Figure 1). Results showed that from 24 h, optimization-CCS (Supplementary Figure 2) and Simpson diversity indices (Supplementary Figure 2) significantly increased, while OUT/Ace/Chao1 (Supplementary Figure 2), Shannon diversity indices (Supplementary Figure 2) decreased when compared to the controls. With the aspects of different treatments, group E decreased optimization-CCS. The maximum percentage of β-diversity variation measured by weighted UniFrac matrices in different experimental groups was 94.62% along PC1, 3.91% along PC2, and 0.51% along PC3 by PCA analysis (*p* < 0.05).

The relative abundances of Aerobic (Actinobacteria, Planctomycetes, Proteobacteria), Anaerobic (Bacteroidetes, Planctomycetes), Contains_Mobile_Elements (Proteobacteria), Facultatively_Anaerobic (Proteobacteria), Forms_Biofilms (Actinobacteria, Planctomycetes, Proteobacteria), Gram_Negative (Planctomycetes, Proteobacteria), Gram_Positive (Actinobacteria), Potentially_Pathogenic (Proteobacteria, TM7), and Stress_Tolerant (Proteobacteria) are revealed in Supplementary Figure 3. The branches of Actinobacteria, Planctomycetes, and Proteobacteria have been revealed as the significant biomarkers based on the total expression in all the treatment groups.

When we compared group C (named M52/53/54) with group D (named M55/56/57) at the class 2 level, the significant 17 COG categories have been enriched in the comparison between groups C and D at 24 h. The categories are ordered as amino acid transport and metabolism, carbohydrate transport and metabolism, coenzyme transport and metabolism, defense mechanisms, inorganic ion transport and metabolism, intracellular trafficking, secretion and vesicular transport, lipid transport and metabolism, secondary metabolites biosynthesis, and transport and catabolism.

In the KEGG pathway at the phylum class 1 level, environmental information processing and metabolism have been enriched. Interestingly, the significant 27 KEGG pathways in the comparison between groups C and D at 24 h, named as biosynthesis of other secondary metabolites, carbohydrate metabolism, energy metabolism, environmental adaptation, glycan biosynthesis and metabolism, and infectious diseases: bacterial, parasitic, and viral, membrane transport, metabolism of other amino acids, metabolism of terpenoids and polyketides, and xenobiotics biodegradation and metabolism.

In group C, when we compared 0 h (chosen M07 as a reference control) and 24 h (chosen M52 as the case), the significant biomarkers were Planctomycetes and Actinobacteria. Al@TCAP enhanced the expression of Pirellulaceae. In group B, when we compared 12 h (i.e., M35) and 48 h (i.e., M81), the expression of Planctomycetes and Proteobacteria increased and decreased in the volcanic rock group (Supplementary Figure 3).

In the network between the significant biomarkers in the process of water purification, the current study found the top 10 were Pirellulaceae and Polynucleobacter. *Filimonas* and *Fluviicola* are mainly involved in the degradation of high-concentration organic matter, while the relative abundance of *Novosphingobium*, *Reyranella*, *Shinella*, *Nevskia*, and *Brevundimonas* (both belong to Proteobacteria) was positively correlated with the content of polycyclic aromatic hydrocarbon analogs (Supplementary Figure 3). In the comparison between groups C and D, the significant KEGG pathway number of metabolism (other secondary metabolites, carbohydrate, energy, glycan, other amino acids, terpenoids, and polyketides), environmental response (xenobiotics biodegradation, adaptation), and disease prevention (infectious diseases: bacterial, parasitic, viral) are revealed in Supplementary Figure 3.

### α/β-diversity for operating taxonomical units *via* ITS-Seq method

Based on the cluster images of species richness, 141 OTUs were collected. Chytridiomycota (71.51%) was the primary group phylum. Ascomycota, Basidiomycota, and Kickxellomycota displayed significant differences between the groups (Supplementary Figure 1). When assessing the samples, seven gates were detected, and there were differences between the collected samples.

Results showed from 48 h, optimization-CCS (Supplementary Figure 2) and Simpson diversity indices (Supplementary Figure 2) significantly increased, while OUT/ACE/Chao1 (Supplementary Figure 2) and Shannon diversity indices significantly increased when compared with those at 6 h. OTU, ACE, Chao1, and Shannon diversity indices at 36 and 48 h significantly decreased when compared with those at 0 h, while Simpson diversity indices significantly increased. With the aspects of different treatments, three purification material addition groups, containing groups B, C, and E decreased optimization-CCS, while the others showed no significant differences when compared to the controls.

The variation in β-diversity measured by Bray–Curtis in different experimental groups, based on an NMDS and Anosim analysis, found that S1, S2, and S3 are similar, and S4, S5, and S6 are extremely different between and within groups, indicating that the microbial communities gradually developed over time (Supplementary Table 1). There was little difference among samples. The maximum percentage of β-diversity variation measured by weighted UniFrac matrices in different experimental groups was 90.18% along PC1, 7.07% along PC2, and 0.88% along PC3 by PCA analysis (*p* < 0.05).

**FIGURE 4:**
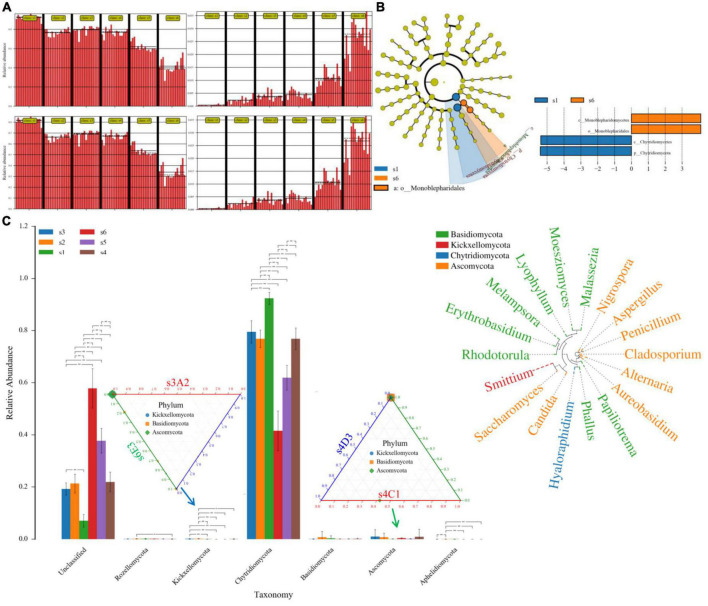
Fungal species-abundance clustering image at different category levels. **(A)**, the significant difference of sample *via* heatmapby binary_jaccard_dm. method; **(B)**, biomarkers *via* the lefse and cladogram method; **(C)**, group ANOVA result in the phylum level, the relative abundance among the comparisons of s4C1 *vs.* s4D3, s3A2 *vs.* s6E3, while the relative abundance of the genus has also been revealed. Ascomycota (*Nigrospora, Aspergillus, Cladosporium, Alternaria, Candida*), Basidiomycota (*Malassezia, Lyophyllum, Phallus*), and Kickxellomycota (*Smittium*) increased. The relationship between the selected taxonomy group (abundant species, genera, classes, orders, or families) was calculated using SPSS 25.0 software.

The decreased Chytridiomycetes (especially *Hyaloraphidium*) and increased Monoblepharidomycetes has been shown in the present study with the increasing time. Except for Basidiomycota, the branch of Aphelidiomycota, Ascomycota, Chytridiomycota, Kickxellomycota, and Rozellomycota showed as the significant biomarker between different time durations *via* ANOVA analysis (Figure 4). At 24 h, when we compared group C (chosen s4C1 as the reference control) and group D (chosen s4D3 as the case), the significant biomarkers belonged to Ascomycota. Kickxellomycota, Basidiomycota, and Ascomycota were the significant biomarkers between group A at 12 h (s3A2) and E at 48 h (s6E3) *via* the Ternary method. Compared with the S2 group, the genus *Aspergillus*, genus *Penicillium*, species *Aspergillus penicillioides*, and species *Penicillium lemhiflumine* in the S5 group were significantly reduced. Compared with the S2 group, the genus *Penicillium* and species *Penicillium lemhiflumine* in the S6 group decreased significantly (Figure 4). Compared with the S3 group, the genus *Aspergillus* in the S5 group was significantly reduced. Compared with the S4 group, the genus *Penicillium* in the S5 group decreased significantly. Compared with the S4 group, the genus *Penicillium* and species *Penicillium lemhiflumin* in the S6 group were significantly reduced. Compared with the S5 group, the genus Aspergillus in the S6 group increased significantly.

## Discussion

### Superficial characteristics and the related nutrient removal efficiency

Attapulgite and volcanic rock exhibit excellent adsorption performance and improve the environmental indicators of water quality (Fan et al., 2011; Derakhshan et al., 2016). It can be seen from the characterization experiments on the surface of the purification material that Al@TCAP has a rough and uneven surface with a large number of protruding, gully-like particles, and pores under the particles. The specific surface area is large, approximately 2.94 and 2.10 times for adsorption and desorption of the volcanic rock, but the microbial capacity on the surface is weak, and no bacterial film is formed. The study demonstrates that Al@TCAP exhibits a weak microbial loading capacity, and the Al@TCAP removal is primarily attributed to adsorption. At 48 h, the removal rates of TN, TP, and COD_Mn_ in the purified materials plus the three exogenous microorganisms were over 47%. The volcanic rock has a sufficient ability to load microorganisms, and the organisms primarily perform the removal for improved water quality.

The exogenous microorganism and activated sludge are often used for water pollution treatment, strengthening the denitrification capacity of the polluted water (Fan et al., 2020). In group E of the present study, the NO_3_^–^-N concentration increased and decreased within 6–12 h and 12–24 h, respectively, and the NO_2_^–^-N concentration continued to grow within 6–24 h, indicating that the nitrification reaction occurred after the actions of exogenous microorganisms. In the original group E, the concentration of NO_3_^–^-N decreased at 12–36 h, and the corresponding 24–48 h NAR increased. The 6–36 h NIR increased, indicating that denitrification continued to increase at 12–36 h and that nitrification and denitrification coexisted at 12–24 h (Kim et al., 2017; Zhang et al., 2020). At 12 h, the proportion of Planctomycetes increased in groups C, D, and E. In comparison, the percentage of Planctomycetes increased at 24 h in groups A and B. This shows that Al@TCAP and exogenous microorganisms accelerated the nitrification reaction and advanced the denitrification reaction (Zhang et al., 2020). In actual production, when aerating can be determined according to the NH_3_-N concentration of the primary precipitation unit, prolonging the nitrification reaction reduces the impact of NH_3_-N on the water body. The fungi detected by ITS-Seq contained Penicillium and Aspergillus, which have been proven to have PO_4_^3–^-P-dissolving functions, and their PO_4_^3–^-P-dissolving ability is typically more reliable than that of bacteria. Proteobacteria contain Nitrosomonas, which can oxidize ammonia (Ilgrande et al., 2018). Planctomycetes include a type of bacteria that can convert NO_2_^–^-N to NH_3_-N to form N_2_. This reaction is called anaerobic ammonium oxidation (Zheng Y. et al., 2019). The current study suggests that those bacteria play a vital role in the nitrogen cycle (Zhang et al., 2020). In addition, the correlation analysis of DO and NAR activities were significantly positively correlated (*p* < 0.05), indicating that increasing DO in water can increase NAR activity. Due to the decrease in DO, the AMO activity of the oxidized NH_3_-N decreased, and NIR reduced NO_2_^––^-N to NH_3_-N, which gradually increased the concentration of NH_3_-N in the water.

In this experiment, at 36 h, the OPH activity of each group was higher than that of the new group. Compared with other periods, the proportion of Penicillium at 36 h was significantly lower, which explains the flat TP removal rate at 24–36 h (Watanabe et al., 2010). The AKP activity and TP removal rate of group E at 48 h were higher than those of the group with the added purification materials and the exogenous microorganisms (Pundir et al., 2019; Zheng L. et al., 2019). The content of the genus Aspergillus at 48 h was higher than at 36 h, indicating that the purification materials assisted in the growth of certain exogenous microorganism environments and improved the growth of microorganisms associated with TP removal (Cheng et al., 2019).

The dehydrogenase activity of each group was higher than that of the control group at 12–24 h, and the COD_Mn_ removal rate of groups D and E with the added exogenous microorganisms was higher than that of the other groups, indicating that exogenous microorganisms can accelerate the initiation of organic matter degradation (Chen et al., 2020) and enhance the removal rate of COD_Mn_.

### Enhance the effect of exogenous microorganisms

In the comparison between groups C and D, the KEGG pathway of metabolism, environmental response, and disease prevention have been significantly affected, while Al@TCAP enhanced the expression of Pirellulaceae in the present study. Otherwise, Al@TCAP increased the expression of Planctomycetes and Actinobacteria. Planctomycetes were the dominant microbes (13% of the microbial community) under anaerobic conditions during the nitrogen cycle (Li et al., 2017). The relative occupied abundances of Proteobacteria offered their use in wastewater treatment, especially the bioremediation of hydrocarbon pollutants (Romero et al., 2019). The volcanic rock increased the expression of Planctomycetes, decreased the expression of Proteobacteria, and a recent study showed that volcanic rock had a retention capacity of heavy metals when combined with the use of biochar (Piscitelli et al., 2018).

Within the fungi in the present study, the purification material addition group decreased the expression of *Hyaloraphidium*, while no available data for *Hyaloraphidium* has been found. The decreased Chytridiomycetes (Hyaloraphidium) and the increased Monoblepharidomycetes were found with the increasing time, especially from 36 to 48 h. Chytridiomycetes was found to be associated with the degradation of organic matter and trace-organic contaminants (Maza-Márquez et al., 2016), the diversity indices of 16SrRNA- (24–48 h), ITS-Seq (36–48 h), and DHO of group C/D decreased at 36–48 h. The present study showed that organic matter degradation declined from 36 h. Ascomycota (*Aspergillus* and *Candida*; Assress et al., 2019) and Basidiomycota were the predominant fungal phylum during the thermophilic phase in the use of sewage sludge (Liu et al., 2019; Robledo-Mahón et al., 2020). Ascomycota, Basidiomycota, and Kickxellomycota increased in group D when compared with B at 24 h, while group E increased Ascomycota at 48 h. Actinobacteria, Proteobacteria, and Aspergillus significantly changed in the biochar applied treatments (Awasthi et al., 2017), Candida was useful for the removal of high-strength phenolic compounds (Basak et al., 2019) and similar to our study, it hinted that purification material addition may have the same effect as biochar in the wastewater treatment (Rocha et al., 2020).

## Conclusion

The ability of Al@TCAP to load microorganisms is weak, and the water quality is predominantly purified by adsorption. The volcanic rock exhibits a strong ability to load microorganisms, and the water quality is primarily purified by microorganisms. At 48 h, the removal rates of TN, TP, and COD_Mn_ by the purification materials plus three exogenous microorganisms were 46.91, 50.93, and 65.08%, respectively. The study found that volcanic rock, Al@TCAP, and exogenous microorganisms can increase the purification effect by increasing the OPH, DHO, and NIR activities in a specific period. The combination of the composite purification material and three types of exogenous microorganisms can increase AKP activity and improve the TP purification effect at 48 h. In the experiment, Al@TCAP and exogenous microorganisms promoted the growth of Planctomycetes and accelerated denitrification. The purification material also provided a growth environment for microorganisms with phosphorus-decomposing functions and enhanced TP removal.

## Data availability statement

The data presented in the study is deposited in the SRA repository (https://www.ncbi.nlm.nih.gov/ PRJNA392982).

## Author contributions

YZ, JY, and GX conceived and designed the experiments. YZ and YW analyzed the data. XY and JG contributed reagents, materials, and analysis tools. YZ contributed to the figures preparation. YZ prepared and wrote the manuscript. All authors reviewed the manuscript.
