# Supplementary material for: Effective mechanisms of water purification for nitrogen-modified attapulgite, volcanic rock, and combined exogenous microorganisms
Source: Front Microbiol. 2022 Aug 10;13:944366. doi: 10.3389/fmicb.2022.944366 (PMC9399813; doi:10.3389/fmicb.2022.944366)
Supplement: Supplementary file 1 [file Data_Sheet_1.docx]

**
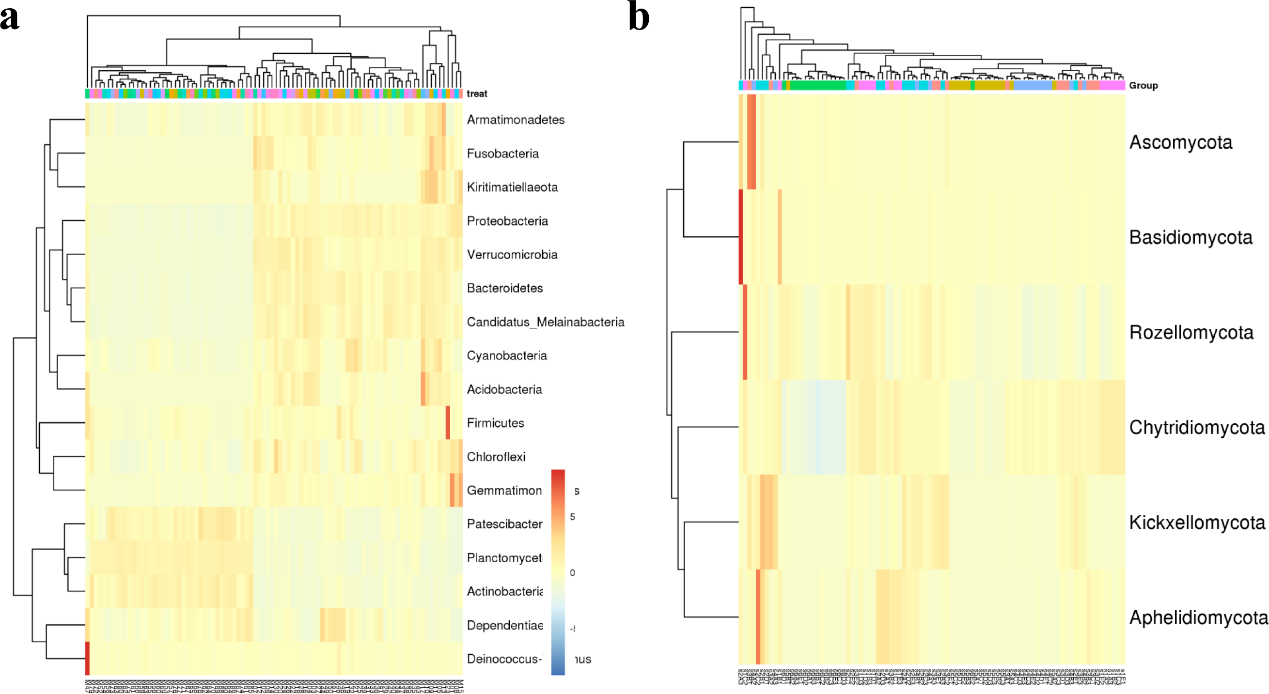
**

**Fig. S1.** The heatmap in the significant phylum level of the bacterium (a) and fungus (b) genera.

**
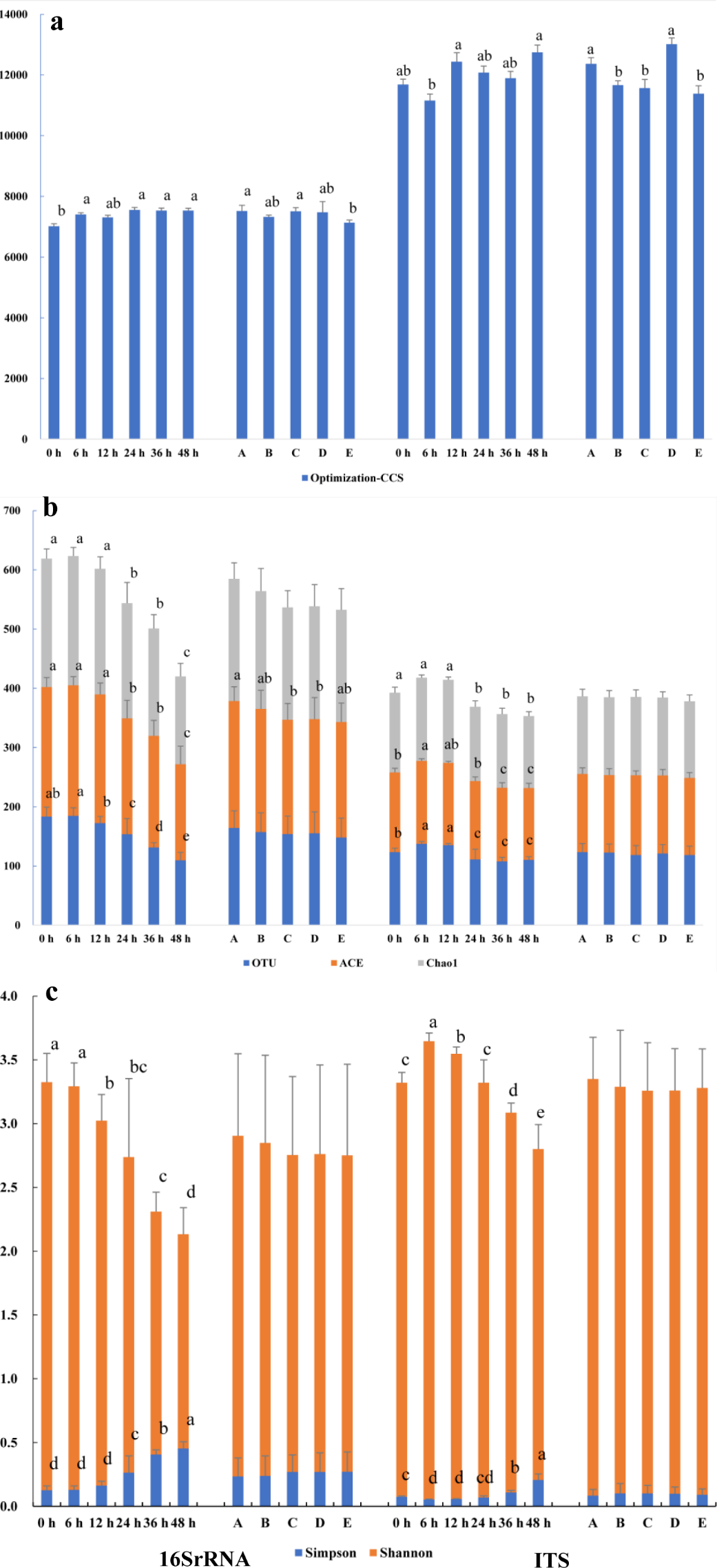
**

**Fig. S2.** The optimization-CCS, OTU, Ace, Chao1, Simpson, and Shannon diversity indices of the bacterial and fungal community between the treatment and control groups. For all parameters (enzyme activity, water quality, 16SrRNA- and ITS-Seq indicators containing optimization-CCS, OTU number, ACE, Chao1, Simpson, Shannon indexes, the expression of the branches via ITS-Seq method), data were compared using a one-way analysis of variance at the end of each bioassay.


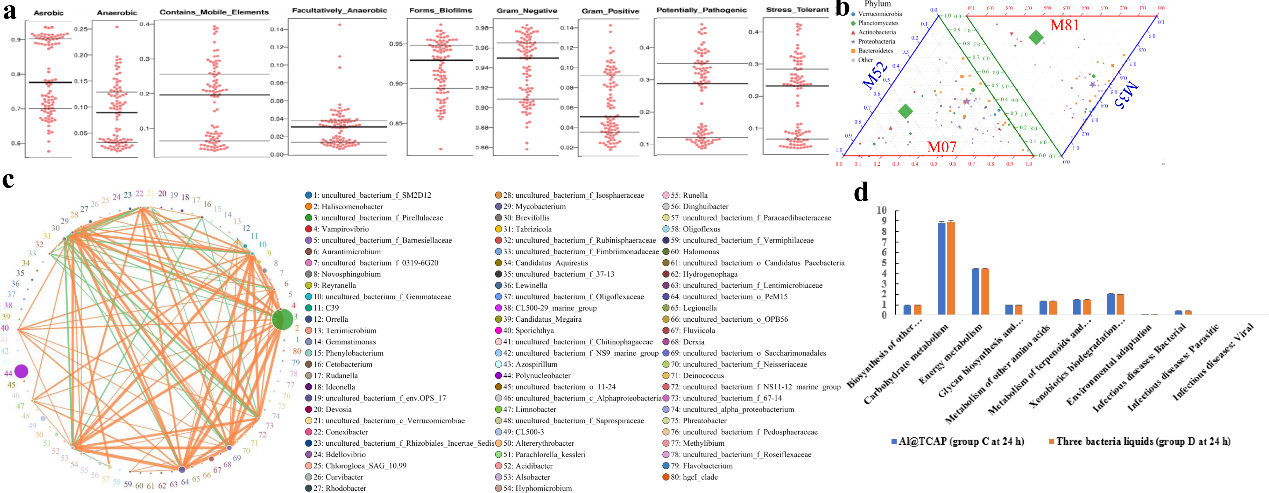


**Fig. S3.** Bacterial species-abundance clustering image at different category levels. a, relative abundances of functional genera; b, the biomarker of the comparison of group C (Al@TCAP) at 24 and 0 h, group B (volcanic rock) at 12 and 48 h; c, network between the significant biomarkers; d, the significant KEGG pathway numbers of metabolism, environmental response and disease prevention in the comparison between Al@TCAP and three bacteria liquids at 24 h.

**Table S1.** The expression of the branches via ITS-Seq method.

| s1 vs s2 |  | Mean(s1) | Mean(s2) | P value |
| --- | --- | --- | --- | --- |
| phylum | Chytridiomycota | 9.24E-01 | 7.68E-01 | 9.99E-04 |
|  | Kickxellomycota | 1.78E-04 | 1.71E-03 | 9.99E-04 |
|  | Ascomycota | 6.88E-04 | 7.38E-03 | 5.99E-03 |
| order | GS10 | 3.84E-04 | 8.49E-04 | 9.99E-04 |
|  | Harpellales | 1.78E-04 | 1.71E-03 | 9.99E-04 |
|  | Malasseziales | 6.51E-05 | 5.04E-04 | 9.99E-04 |
|  | Monoblepharidales | 3.89E-04 | 2.35E-03 | 9.99E-04 |
|  | Rhizophydiales | 2.21E-03 | 3.81E-03 | 3.00E-03 |
|  | Pleosporales | 4.45E-05 | 3.43E-04 | 7.99E-03 |
|  | Eurotiales | 2.02E-05 | 2.98E-04 | 1.50E-02 |
|  | Hypocreales | 1.31E-05 | 6.23E-05 | 1.98E-02 |
|  | Ustilaginales | 2.73E-05 | 7.84E-05 | 2.77E-02 |
|  | Dothideales | 2.89E-05 | 2.23E-04 | 3.20E-02 |
|  | Saccharomycetales | 7.67E-05 | 1.72E-03 | 3.80E-02 |
| s1 vs s3 |  | Mean(s1) | Mean(s3) | P value |
| class | Chytridiomycetes | 8.15E-01 | 6.88E-01 | 9.99E-04 |
|  | Cystobasidiomycetes | 0.00E+00 | 2.72E-04 | 9.99E-04 |
|  | Harpellomycetes | 1.78E-04 | 1.23E-03 | 9.99E-04 |
|  | Malasseziomycetes | 6.51E-05 | 3.85E-04 | 9.99E-04 |
|  | Monoblepharidomycetes | 3.89E-04 | 3.75E-03 | 9.99E-04 |
|  | Rozellomycotina_cls_Incertae_sedis | 3.84E-04 | 7.50E-04 | 2.00E-03 |
|  | Saccharomycetes | 7.67E-05 | 9.78E-04 | 5.99E-03 |
|  | Dothideomycetes | 5.42E-04 | 1.18E-03 | 1.80E-02 |
|  | Tremellomycetes | 4.68E-05 | 4.61E-06 | 3.36E-02 |
| order | Erythrobasidiales | 0.00E+00 | 2.72E-04 | 9.99E-04 |
|  | GS10 | 3.84E-04 | 7.50E-04 | 9.99E-04 |
|  | Monoblepharidales | 3.89E-04 | 3.75E-03 | 9.99E-04 |
|  | Harpellales | 1.78E-04 | 1.23E-03 | 2.00E-03 |
|  | Malasseziales | 6.51E-05 | 3.85E-04 | 2.00E-03 |
|  | Pleosporales | 4.45E-05 | 4.14E-04 | 2.00E-03 |
|  | Saccharomycetales | 7.67E-05 | 9.78E-04 | 4.00E-03 |
|  | Hypocreales | 1.31E-05 | 6.63E-05 | 4.49E-03 |
|  | Rhizophydiales | 2.21E-03 | 3.26E-03 | 2.80E-02 |
|  | Tremellales | 4.68E-05 | 4.61E-06 | 3.36E-02 |
| family | Erythrobasidiaceae | 0.00E+00 | 2.72E-04 | 9.99E-04 |
|  | Legeriomycetaceae | 1.78E-04 | 1.23E-03 | 9.99E-04 |
|  | Malasseziaceae | 6.51E-05 | 3.85E-04 | 9.99E-04 |
|  | Saccharomycetaceae | 0.00E+00 | 4.26E-04 | 9.99E-04 |
|  | Pleosporaceae | 4.45E-05 | 4.14E-04 | 4.00E-03 |
|  | Cordycipitaceae | 1.31E-05 | 6.63E-05 | 4.49E-03 |
|  | Debaryomycetaceae | 7.67E-05 | 5.52E-04 | 2.50E-02 |
|  | Rhynchogastremataceae | 4.68E-05 | 4.61E-06 | 3.36E-02 |
| genus | *Erythrobasidium* | 0.00E+00 | 2.72E-04 | 9.99E-04 |
|  | *Hyaloraphidium* | 3.89E-04 | 3.75E-03 | 9.99E-04 |
|  | *Saccharomyces* | 0.00E+00 | 4.26E-04 | 9.99E-04 |
|  | *Smittium* | 1.78E-04 | 1.23E-03 | 9.99E-04 |
|  | *Malassezia* | 6.51E-05 | 3.85E-04 | 2.00E-03 |
|  | *Alternaria* | 4.45E-05 | 4.14E-04 | 3.00E-03 |
|  | *Candida* | 7.67E-05 | 5.52E-04 | 1.80E-02 |
|  | *Papiliotrema* | 4.68E-05 | 4.61E-06 | 3.36E-02 |
| species | *Erythrobasidium_hasegawianum* | 0.00E+00 | 2.72E-04 | 9.99E-04 |
|  | *Hyaloraphidium_curvatum* | 3.89E-04 | 3.75E-03 | 9.99E-04 |
|  | *Malassezia_restricta* | 6.51E-05 | 3.85E-04 | 9.99E-04 |
|  | *Saccharomyces_mikatae* | 0.00E+00 | 4.26E-04 | 9.99E-04 |
|  | *Smittium_morbosum* | 1.78E-04 | 1.23E-03 | 2.00E-03 |
|  | *Alternaria_destruens* | 4.45E-05 | 4.14E-04 | 3.00E-03 |
|  | *Candida_tropicalis* | 4.44E-05 | 5.38E-04 | 1.30E-02 |
|  | *Papiliotrema_flavescens* | 4.68E-05 | 4.61E-06 | 3.36E-02 |
| s1 vs s4 |  | Mean(s1) | Mean(s4) | P value |
| phylum | Chytridiomycota | 9.24E-01 | 7.68E-01 | 9.99E-04 |
|  | Aphelidiomycota | 1.93E-04 | 5.63E-05 | 2.20E-02 |
|  | Rozellomycota | 1.37E-03 | 6.29E-04 | 4.30E-02 |
|  | Kickxellomycota | 1.78E-04 | 6.93E-04 | 4.40E-02 |
| class | Chytridiomycetes | 8.15E-01 | 6.69E-01 | 9.99E-04 |
|  | Cystobasidiomycetes | 0.00E+00 | 1.87E-04 | 9.99E-04 |
|  | Monoblepharidomycetes | 3.89E-04 | 4.44E-03 | 9.99E-04 |
|  | Sordariomycetes | 4.91E-05 | 2.29E-04 | 9.99E-04 |
|  | Agaricomycetes | 2.86E-03 | 6.92E-06 | 2.00E-03 |
|  | Malasseziomycetes | 6.51E-05 | 5.53E-04 | 2.40E-02 |
|  | Harpellomycetes | 1.78E-04 | 6.93E-04 | 4.80E-02 |
| genus | *Erythrobasidium* | 0.00E+00 | 1.87E-04 | 9.99E-04 |
|  | *Hyaloraphidium* | 3.89E-04 | 4.44E-03 | 9.99E-04 |
|  | *Lyophyllum* | 1.24E-03 | 6.92E-06 | 9.99E-04 |
|  | *Penicillium* | 0.00E+00 | 2.66E-04 | 9.99E-04 |
|  | *Phallus* | 1.61E-03 | 0.00E+00 | 9.99E-04 |
|  | *Nigrospora* | 3.59E-05 | 1.54E-04 | 5.99E-03 |
|  | *Saccharomyces* | 0.00E+00 | 4.18E-05 | 1.58E-02 |
|  | *Malassezia* | 6.51E-05 | 5.53E-04 | 2.20E-02 |
|  | *Smittium* | 1.78E-04 | 6.93E-04 | 3.90E-02 |
| species | *Erythrobasidium_hasegawianum* | 0.00E+00 | 1.87E-04 | 9.99E-04 |
|  | *Hyaloraphidium_curvatum* | 3.89E-04 | 4.44E-03 | 9.99E-04 |
|  | *Penicillium_lemhiflumine* | 0.00E+00 | 2.66E-04 | 9.99E-04 |
|  | *Phallus_haitangensis* | 1.61E-03 | 0.00E+00 | 9.99E-04 |
|  | *Lyophyllum_fumosum* | 1.24E-03 | 6.92E-06 | 2.00E-03 |
|  | *Nigrospora_zimmermanii* | 3.59E-05 | 1.54E-04 | 4.00E-03 |
|  | *Saccharomyces_mikatae* | 0.00E+00 | 4.18E-05 | 1.58E-02 |
|  | *Malassezia_restricta* | 6.51E-05 | 5.53E-04 | 2.70E-02 |
| s1 vs s5 |  | Mean(s1) | Mean(s5) | P value |
| phylum | Aphelidiomycota | 1.93E-04 | 0.00E+00 | 9.99E-04 |
|  | Chytridiomycota | 9.24E-01 | 6.20E-01 | 9.99E-04 |
|  | Kickxellomycota | 1.78E-04 | 1.88E-05 | 3.00E-03 |
|  | Ascomycota | 6.88E-04 | 1.45E-03 | 9.99E-03 |
| order | Agaricales | 1.24E-03 | 5.38E-06 | 9.99E-04 |
|  | Erythrobasidiales | 0.00E+00 | 8.20E-05 | 9.99E-04 |
|  | GS10 | 3.84E-04 | 6.97E-04 | 9.99E-04 |
|  | Harpellales | 1.78E-04 | 1.88E-05 | 9.99E-04 |
|  | Malasseziales | 6.51E-05 | 3.46E-04 | 9.99E-04 |
|  | Monoblepharidales | 3.89E-04 | 1.07E-02 | 9.99E-04 |
|  | Phallales | 1.61E-03 | 0.00E+00 | 9.99E-04 |
|  | Rhizophydiales | 2.21E-03 | 2.96E-04 | 9.99E-04 |
|  | Trichosphaeriales | 3.59E-05 | 4.14E-04 | 9.99E-04 |
|  | Tremellales | 4.68E-05 | 0.00E+00 | 7.06E-03 |
|  | Pleosporales | 4.45E-05 | 2.53E-04 | 7.99E-03 |
|  | Pucciniales | 1.27E-05 | 1.77E-04 | 2.70E-02 |
| family | Erythrobasidiaceae | 0.00E+00 | 8.20E-05 | 9.99E-04 |
|  | Legeriomycetaceae | 1.78E-04 | 1.88E-05 | 9.99E-04 |
|  | Malasseziaceae | 6.51E-05 | 3.46E-04 | 9.99E-04 |
|  | Phallaceae | 1.61E-03 | 0.00E+00 | 9.99E-04 |
|  | Trichosphaeriaceae | 3.59E-05 | 4.14E-04 | 9.99E-04 |
|  | Pleosporaceae | 4.45E-05 | 2.53E-04 | 2.00E-03 |
|  | Lyophyllaceae | 1.24E-03 | 5.38E-06 | 4.00E-03 |
|  | Rhynchogastremataceae | 4.68E-05 | 0.00E+00 | 7.06E-03 |
| species | *Erythrobasidium_hasegawianum* | 0.00E+00 | 8.20E-05 | 9.99E-04 |
|  | *Hyaloraphidium_curvatum* | 3.89E-04 | 1.07E-02 | 9.99E-04 |
|  | *Lyophyllum_fumosum* | 1.24E-03 | 5.38E-06 | 9.99E-04 |
|  | *Malassezia_restricta* | 6.51E-05 | 3.46E-04 | 9.99E-04 |
|  | *Nigrospora_zimmermanii* | 3.59E-05 | 4.14E-04 | 9.99E-04 |
|  | *Phallus_haitangensis* | 1.61E-03 | 0.00E+00 | 9.99E-04 |
|  | *Smittium_morbosum* | 1.78E-04 | 1.88E-05 | 2.00E-03 |
|  | *Alternaria_destruens* | 4.45E-05 | 2.53E-04 | 3.00E-03 |
|  | *Papiliotrema_flavescens* | 4.68E-05 | 0.00E+00 | 7.06E-03 |
|  | *Candida_railenensis* | 3.23E-05 | 0.00E+00 | 2.91E-02 |
|  | *Melampsora_chelidonii-pierotii* | 1.27E-05 | 1.77E-04 | 3.00E-02 |
| s1 vs s6 |  | Mean(s1) | Mean(s6) | P value |
| phylum | Aphelidiomycota | 1.93E-04 | 1.28E-05 | 9.99E-04 |
|  | Ascomycota | 6.88E-04 | 3.77E-03 | 9.99E-04 |
|  | Chytridiomycota | 9.24E-01 | 4.16E-01 | 9.99E-04 |
|  | Kickxellomycota | 1.78E-04 | 2.27E-05 | 5.99E-03 |
| class | Agaricomycetes | 2.86E-03 | 0.00E+00 | 9.99E-04 |
|  | Chytridiomycetes | 8.15E-01 | 3.39E-01 | 9.99E-04 |
|  | Dothideomycetes | 5.42E-04 | 2.00E-03 | 9.99E-04 |
|  | Monoblepharidomycetes | 3.89E-04 | 2.68E-02 | 9.99E-04 |
|  | Rozellomycotina_cls_Incertae_sedis | 3.84E-04 | 9.63E-04 | 9.99E-04 |
|  | Sordariomycetes | 4.91E-05 | 1.14E-03 | 9.99E-04 |
|  | Malasseziomycetes | 6.51E-05 | 3.43E-04 | 2.00E-03 |
|  | Harpellomycetes | 1.78E-04 | 2.27E-05 | 4.00E-03 |
|  | Cystobasidiomycetes | 0.00E+00 | 5.39E-05 | 4.29E-03 |
|  | Ustilaginomycetes | 2.73E-05 | 2.14E-04 | 3.80E-02 |
| family | Cladosporiaceae | 4.68E-04 | 1.55E-03 | 9.99E-04 |
|  | Lyophyllaceae | 1.24E-03 | 0.00E+00 | 9.99E-04 |
|  | Malasseziaceae | 6.51E-05 | 3.43E-04 | 9.99E-04 |
|  | Phallaceae | 1.61E-03 | 0.00E+00 | 9.99E-04 |
|  | Trichosphaeriaceae | 3.59E-05 | 9.94E-04 | 9.99E-04 |
|  | Pleosporaceae | 4.45E-05 | 4.12E-04 | 2.00E-03 |
|  | Legeriomycetaceae | 1.78E-04 | 2.27E-05 | 3.00E-03 |
|  | Cordycipitaceae | 1.31E-05 | 1.45E-04 | 4.00E-03 |
|  | Erythrobasidiaceae | 0.00E+00 | 5.39E-05 | 4.29E-03 |
|  | Ustilaginaceae | 2.73E-05 | 2.14E-04 | 3.30E-02 |
|  | Melampsoraceae | 1.27E-05 | 8.17E-05 | 3.90E-02 |
| genus | *Alternaria* | 4.45E-05 | 4.12E-04 | 9.99E-04 |
|  | *Cladosporium* | 4.68E-04 | 1.55E-03 | 9.99E-04 |
|  | *Hyaloraphidium* | 3.89E-04 | 2.68E-02 | 9.99E-04 |
|  | *Lyophyllum* | 1.24E-03 | 0.00E+00 | 9.99E-04 |
|  | *Malassezia* | 6.51E-05 | 3.43E-04 | 9.99E-04 |
|  | *Nigrospora* | 3.59E-05 | 9.94E-04 | 9.99E-04 |
|  | *Phallus* | 1.61E-03 | 0.00E+00 | 9.99E-04 |
|  | *Smittium* | 1.78E-04 | 2.27E-05 | 3.00E-03 |
|  | *Erythrobasidium* | 0.00E+00 | 5.39E-05 | 4.29E-03 |
|  | *Moesziomyces* | 2.73E-05 | 2.14E-04 | 2.80E-02 |
|  | *Melampsora* | 1.27E-05 | 8.17E-05 | 3.90E-02 |
| s2 vs s3 |  | Mean(s2) | Mean(s3) | P value |
| class | Tremellomycetes | 7.76E-05 | 4.61E-06 | 5.82E-04 |
|  | Ustilaginomycetes | 7.84E-05 | 8.31E-06 | 2.91E-03 |
|  | Monoblepharidomycetes | 2.35E-03 | 3.75E-03 | 5.00E-03 |
|  | Chytridiomycetes | 6.63E-01 | 6.88E-01 | 4.50E-02 |
| order | Tremellales | 7.76E-05 | 4.61E-06 | 5.82E-04 |
|  | Ustilaginales | 7.84E-05 | 8.31E-06 | 2.91E-03 |
|  | Monoblepharidales | 2.35E-03 | 3.75E-03 | 4.00E-03 |
|  | Dothideales | 2.23E-04 | 2.18E-05 | 2.40E-02 |
| species | *Papiliotrema_flavescens* | 7.76E-05 | 4.61E-06 | 5.82E-04 |
|  | *Moesziomyces_aphidis* | 7.84E-05 | 8.31E-06 | 2.91E-03 |
|  | *Hyaloraphidium_curvatum* | 2.35E-03 | 3.75E-03 | 5.99E-03 |
|  | *Aureobasidium_melanogenum* | 2.23E-04 | 2.18E-05 | 2.60E-02 |
| s2 vs s4 |  | Mean(s2) | Mean(s4) | P value |
| phylum | Aphelidiomycota | 3.88E-04 | 5.63E-05 | 9.99E-04 |
|  | Rozellomycota | 1.40E-03 | 6.29E-04 | 9.99E-04 |
|  | Kickxellomycota | 1.71E-03 | 6.93E-04 | 1.90E-02 |
| class | Monoblepharidomycetes | 2.35E-03 | 4.44E-03 | 9.99E-04 |
|  | Pucciniomycetes | 0.00E+00 | 4.26E-05 | 8.24E-03 |
|  | Agaricomycetes | 6.28E-03 | 6.92E-06 | 9.99E-03 |
|  | Harpellomycetes | 1.71E-03 | 6.93E-04 | 2.40E-02 |
|  | Rozellomycotina_cls_Incertae_sedis | 8.49E-04 | 5.32E-04 | 3.50E-02 |
| order | Phallales | 2.98E-03 | 0.00E+00 | 9.99E-04 |
|  | Rhizophydiales | 3.81E-03 | 1.50E-03 | 9.99E-04 |
|  | Monoblepharidales | 2.35E-03 | 4.44E-03 | 2.00E-03 |
|  | Pucciniales | 0.00E+00 | 4.26E-05 | 8.24E-03 |
|  | Harpellales | 1.71E-03 | 6.93E-04 | 1.40E-02 |
|  | GS10 | 8.49E-04 | 5.32E-04 | 3.60E-02 |
| family | Phallaceae | 2.98E-03 | 0.00E+00 | 9.99E-04 |
|  | Melampsoraceae | 0.00E+00 | 4.26E-05 | 8.24E-03 |
|  | Legeriomycetaceae | 1.71E-03 | 6.93E-04 | 2.10E-02 |
| species | *Phallus_haitangensis* | 2.98E-03 | 0.00E+00 | 9.99E-04 |
|  | *Hyaloraphidium_curvatum* | 2.35E-03 | 4.44E-03 | 2.00E-03 |
|  | *Melampsora_chelidonii-pierotii* | 0.00E+00 | 4.26E-05 | 8.24E-03 |
|  | *Smittium_morbosum* | 1.71E-03 | 6.93E-04 | 2.60E-02 |
| s2 vs s5 |  | Mean(s2) | Mean(s5) | P value |
| phylum | Aphelidiomycota | 3.88E-04 | 0.00E+00 | 9.99E-04 |
|  | Chytridiomycota | 7.68E-01 | 6.20E-01 | 9.99E-04 |
|  | Kickxellomycota | 1.71E-03 | 1.88E-05 | 9.99E-04 |
|  | Rozellomycota | 1.40E-03 | 7.71E-04 | 2.00E-03 |
| class | Tremellomycetes | 7.76E-05 | 0.00E+00 | 7.37E-05 |
|  | Chytridiomycetes | 6.63E-01 | 5.36E-01 | 9.99E-04 |
|  | Eurotiomycetes | 2.98E-04 | 0.00E+00 | 9.99E-04 |
|  | Harpellomycetes | 1.71E-03 | 1.88E-05 | 9.99E-04 |
|  | Microbotryomycetes | 8.59E-05 | 0.00E+00 | 9.99E-04 |
|  | Monoblepharidomycetes | 2.35E-03 | 1.07E-02 | 9.99E-04 |
|  | Pucciniomycetes | 0.00E+00 | 1.77E-04 | 9.99E-04 |
|  | Agaricomycetes | 6.28E-03 | 5.38E-06 | 3.00E-03 |
| order | Tremellales | 7.76E-05 | 0.00E+00 | 7.37E-05 |
|  | Eurotiales | 2.98E-04 | 0.00E+00 | 9.99E-04 |
|  | Harpellales | 1.71E-03 | 1.88E-05 | 9.99E-04 |
|  | Monoblepharidales | 2.35E-03 | 1.07E-02 | 9.99E-04 |
|  | Phallales | 2.98E-03 | 0.00E+00 | 9.99E-04 |
|  | Pucciniales | 0.00E+00 | 1.77E-04 | 9.99E-04 |
|  | Rhizophydiales | 3.81E-03 | 2.96E-04 | 9.99E-04 |
|  | Sporidiobolales | 8.59E-05 | 0.00E+00 | 9.99E-04 |
|  | Hypocreales | 6.23E-05 | 5.38E-06 | 4.84E-03 |
|  | Agaricales | 3.31E-03 | 5.38E-06 | 1.10E-02 |
|  | Dothideales | 2.23E-04 | 1.63E-05 | 1.10E-02 |
| family | Rhynchogastremataceae | 7.76E-05 | 0.00E+00 | 7.37E-05 |
|  | Aspergillaceae | 2.98E-04 | 0.00E+00 | 9.99E-04 |
|  | Legeriomycetaceae | 1.71E-03 | 1.88E-05 | 9.99E-04 |
|  | Melampsoraceae | 0.00E+00 | 1.77E-04 | 9.99E-04 |
|  | Phallaceae | 2.98E-03 | 0.00E+00 | 9.99E-04 |
|  | Sporidiobolaceae | 8.59E-05 | 0.00E+00 | 9.99E-04 |
|  | Cordycipitaceae | 6.23E-05 | 5.38E-06 | 4.84E-03 |
|  | Saccotheciaceae | 2.23E-04 | 1.63E-05 | 1.30E-02 |
|  | Lyophyllaceae | 3.31E-03 | 5.38E-06 | 2.00E-02 |
| genus | *Papiliotrema* | 7.76E-05 | 0.00E+00 | 7.37E-05 |
|  | *Aspergillus* | 1.76E-04 | 0.00E+00 | 9.99E-04 |
|  | *Hyaloraphidium* | 2.35E-03 | 1.07E-02 | 9.99E-04 |
|  | *Melampsora* | 0.00E+00 | 1.77E-04 | 9.99E-04 |
|  | *Penicillium* | 1.21E-04 | 0.00E+00 | 9.99E-04 |
|  | *Phallus* | 2.98E-03 | 0.00E+00 | 9.99E-04 |
|  | *Rhodotorula* | 8.59E-05 | 0.00E+00 | 9.99E-04 |
|  | *Smittium* | 1.71E-03 | 1.88E-05 | 9.99E-04 |
|  | *Lyophyllum* | 3.31E-03 | 5.38E-06 | 1.50E-02 |
|  | *Aureobasidium* | 2.23E-04 | 1.63E-05 | 1.60E-02 |
| species | *Papiliotrema_flavescens* | 7.76E-05 | 0.00E+00 | 7.37E-05 |
|  | *Aspergillus_penicillioides* | 1.76E-04 | 0.00E+00 | 9.99E-04 |
|  | *Candida_railenensis* | 1.57E-03 | 0.00E+00 | 9.99E-04 |
|  | *Hyaloraphidium_curvatum* | 2.35E-03 | 1.07E-02 | 9.99E-04 |
|  | *Melampsora_chelidonii-pierotii* | 0.00E+00 | 1.77E-04 | 9.99E-04 |
|  | *Penicillium_lemhiflumine* | 1.21E-04 | 0.00E+00 | 9.99E-04 |
|  | *Phallus_haitangensis* | 2.98E-03 | 0.00E+00 | 9.99E-04 |
|  | *Rhodotorula_babjevae* | 8.59E-05 | 0.00E+00 | 9.99E-04 |
|  | *Smittium_morbosum* | 1.71E-03 | 1.88E-05 | 9.99E-04 |
|  | *Lyophyllum_fumosum* | 3.31E-03 | 5.38E-06 | 8.99E-03 |
|  | *Aureobasidium_melanogenum* | 2.23E-04 | 1.63E-05 | 1.60E-02 |
| s2 vs s6 |  | Mean(s2) | Mean(s6) | P value |
| class | Agaricomycetes | 6.28E-03 | 0.00E+00 | 9.99E-04 |
|  | Chytridiomycetes | 6.63E-01 | 3.39E-01 | 9.99E-04 |
|  | Harpellomycetes | 1.71E-03 | 2.27E-05 | 9.99E-04 |
|  | Monoblepharidomycetes | 2.35E-03 | 2.68E-02 | 9.99E-04 |
|  | Pucciniomycetes | 0.00E+00 | 8.17E-05 | 9.99E-04 |
|  | Sordariomycetes | 1.91E-04 | 1.14E-03 | 9.99E-04 |
| order | Agaricales | 3.31E-03 | 0.00E+00 | 9.99E-04 |
|  | Harpellales | 1.71E-03 | 2.27E-05 | 9.99E-04 |
|  | Monoblepharidales | 2.35E-03 | 2.68E-02 | 9.99E-04 |
|  | Phallales | 2.98E-03 | 0.00E+00 | 9.99E-04 |
|  | Pucciniales | 0.00E+00 | 8.17E-05 | 9.99E-04 |
|  | Rhizophydiales | 3.81E-03 | 5.53E-04 | 9.99E-04 |
|  | Trichosphaeriales | 1.28E-04 | 9.94E-04 | 9.99E-04 |
| genus | *Hyaloraphidium* | 2.35E-03 | 2.68E-02 | 9.99E-04 |
|  | *Lyophyllum* | 3.31E-03 | 0.00E+00 | 9.99E-04 |
|  | *Melampsora* | 0.00E+00 | 8.17E-05 | 9.99E-04 |
|  | *Nigrospora* | 1.28E-04 | 9.94E-04 | 9.99E-04 |
|  | *Penicillium* | 1.21E-04 | 0.00E+00 | 9.99E-04 |
|  | *Phallus* | 2.98E-03 | 0.00E+00 | 9.99E-04 |
|  | *Smittium* | 1.71E-03 | 2.27E-05 | 9.99E-04 |
| species | *Candida_railenensis* | 1.57E-03 | 0.00E+00 | 9.99E-04 |
|  | *Hyaloraphidium_curvatum* | 2.35E-03 | 2.68E-02 | 9.99E-04 |
|  | *Lyophyllum_fumosum* | 3.31E-03 | 0.00E+00 | 9.99E-04 |
|  | *Melampsora_chelidonii-pierotii* | 0.00E+00 | 8.17E-05 | 9.99E-04 |
|  | *Penicillium_lemhiflumine* | 1.21E-04 | 0.00E+00 | 9.99E-04 |
|  | *Phallus_haitangensis* | 2.98E-03 | 0.00E+00 | 9.99E-04 |
|  | *Smittium_morbosum* | 1.71E-03 | 2.27E-05 | 9.99E-04 |
|  | *Nigrospora_zimmermanii* | 1.28E-04 | 9.94E-04 | 2.00E-03 |
|  | *Cladosporium_endophytica* | 6.33E-05 | 1.37E-05 | 2.81E-02 |
|  | *Aureobasidium_melanogenum* | 2.23E-04 | 3.32E-05 | 4.90E-02 |
| s3 vs s4 |  | Mean(s3) | Mean(s4) | P value |
| order | Rhizophydiales | 3.26E-03 | 1.50E-03 | 5.00E-03 |
|  | Agaricales | 3.75E-05 | 6.92E-06 | 3.92E-02 |
| family | Lyophyllaceae | 3.75E-05 | 6.92E-06 | 3.92E-02 |
| species | Lyophyllum_fumosum | 3.75E-05 | 6.92E-06 | 3.92E-02 |
| s3 vs s5 |  | Mean(s3) | Mean(s5) | P value |
| phylum | Aphelidiomycota | 1.23E-04 | 0.00E+00 | 9.99E-04 |
|  | Chytridiomycota | 7.95E-01 | 6.20E-01 | 9.99E-04 |
|  | Kickxellomycota | 1.23E-03 | 1.88E-05 | 9.99E-04 |
| class | Chytridiomycetes | 6.88E-01 | 5.36E-01 | 9.99E-04 |
|  | Eurotiomycetes | 7.39E-03 | 0.00E+00 | 9.99E-04 |
|  | Harpellomycetes | 1.23E-03 | 1.88E-05 | 9.99E-04 |
|  | Monoblepharidomycetes | 3.75E-03 | 1.07E-02 | 9.99E-04 |
|  | Agaricomycetes | 5.00E-05 | 5.38E-06 | 6.45E-03 |
|  | Sordariomycetes | 1.45E-04 | 4.19E-04 | 4.70E-02 |
| family | Aspergillaceae | 7.39E-03 | 0.00E+00 | 9.99E-04 |
|  | Legeriomycetaceae | 1.23E-03 | 1.88E-05 | 9.99E-04 |
|  | Saccharomycetaceae | 4.26E-04 | 0.00E+00 | 9.99E-04 |
|  | Cordycipitaceae | 6.63E-05 | 5.38E-06 | 1.00E-03 |
|  | Trichosphaeriaceae | 7.87E-05 | 4.14E-04 | 1.50E-02 |
|  | Lyophyllaceae | 3.75E-05 | 5.38E-06 | 3.94E-02 |
| genus | *Aspergillus* | 7.36E-03 | 0.00E+00 | 9.99E-04 |
|  | *Hyaloraphidium* | 3.75E-03 | 1.07E-02 | 9.99E-04 |
|  | *Saccharomyces* | 4.26E-04 | 0.00E+00 | 9.99E-04 |
|  | *Smittium* | 1.23E-03 | 1.88E-05 | 9.99E-04 |
|  | *Nigrospora* | 7.87E-05 | 4.14E-04 | 1.70E-02 |
|  | *Lyophyllum* | 3.75E-05 | 5.38E-06 | 3.94E-02 |
| s3 vs s6 |  | Mean(s3) | Mean(s6) | P value |
| phylum | Chytridiomycota | 7.95E-01 | 4.16E-01 | 9.99E-04 |
|  | Kickxellomycota | 1.23E-03 | 2.27E-05 | 9.99E-04 |
|  | Aphelidiomycota | 1.23E-04 | 1.28E-05 | 5.00E-03 |
| order | Harpellales | 1.23E-03 | 2.27E-05 | 9.99E-04 |
|  | Monoblepharidales | 3.75E-03 | 2.68E-02 | 9.99E-04 |
|  | Rhizophydiales | 3.26E-03 | 5.53E-04 | 9.99E-04 |
|  | Trichosphaeriales | 7.87E-05 | 9.94E-04 | 9.99E-04 |
|  | Agaricales | 3.75E-05 | 0.00E+00 | 3.35E-03 |
|  | Ustilaginales | 8.31E-06 | 2.14E-04 | 5.00E-03 |
|  | Capnodiales | 7.48E-04 | 1.55E-03 | 5.99E-03 |
| family | Legeriomycetaceae | 1.23E-03 | 2.27E-05 | 9.99E-04 |
|  | Saccharomycetaceae | 4.26E-04 | 0.00E+00 | 9.99E-04 |
|  | Trichosphaeriaceae | 7.87E-05 | 9.94E-04 | 9.99E-04 |
|  | Ustilaginaceae | 8.31E-06 | 2.14E-04 | 3.00E-03 |
|  | Lyophyllaceae | 3.75E-05 | 0.00E+00 | 3.35E-03 |
|  | Cladosporiaceae | 7.48E-04 | 1.55E-03 | 6.99E-03 |
| genus | *Hyaloraphidium* | 3.75E-03 | 2.68E-02 | 9.99E-04 |
|  | *Nigrospora* | 7.87E-05 | 9.94E-04 | 9.99E-04 |
|  | *Saccharomyces* | 4.26E-04 | 0.00E+00 | 9.99E-04 |
|  | *Smittium* | 1.23E-03 | 2.27E-05 | 9.99E-04 |
|  | *Lyophyllum* | 3.75E-05 | 0.00E+00 | 3.35E-03 |
|  | *Moesziomyces* | 8.31E-06 | 2.14E-04 | 5.00E-03 |
|  | *Cladosporium* | 7.48E-04 | 1.55E-03 | 6.99E-03 |
| species | *Hyaloraphidium_curvatum* | 3.75E-03 | 2.68E-02 | 9.99E-04 |
|  | *Nigrospora_zimmermanii* | 7.87E-05 | 9.94E-04 | 9.99E-04 |
|  | *Saccharomyces_mikatae* | 4.26E-04 | 0.00E+00 | 9.99E-04 |
|  | *Smittium_morbosum* | 1.23E-03 | 2.27E-05 | 9.99E-04 |
|  | *Lyophyllum_fumosum* | 3.75E-05 | 0.00E+00 | 3.35E-03 |
|  | *Moesziomyces_aphidis* | 8.31E-06 | 2.14E-04 | 4.00E-03 |
|  | *Cladosporium_puyae* | 7.06E-04 | 1.54E-03 | 6.99E-03 |
| s4 vs s5 |  | Mean(s4) | Mean(s5) | P value |
| class | Chytridiomycetes | 6.69E-01 | 5.36E-01 | 9.99E-04 |
|  | Eurotiomycetes | 2.90E-04 | 0.00E+00 | 9.99E-04 |
|  | Microbotryomycetes | 2.51E-04 | 0.00E+00 | 9.99E-04 |
|  | Monoblepharidomycetes | 4.44E-03 | 1.07E-02 | 9.99E-04 |
|  | Tremellomycetes | 1.38E-04 | 0.00E+00 | 9.99E-04 |
|  | Harpellomycetes | 6.93E-04 | 1.88E-05 | 5.99E-03 |
| order | Hypocreales | 7.42E-05 | 5.38E-06 | 9.79E-04 |
|  | Eurotiales | 2.90E-04 | 0.00E+00 | 9.99E-04 |
|  | Monoblepharidales | 4.44E-03 | 1.07E-02 | 9.99E-04 |
|  | Sporidiobolales | 2.51E-04 | 0.00E+00 | 9.99E-04 |
|  | Tremellales | 1.38E-04 | 0.00E+00 | 9.99E-04 |
|  | Harpellales | 6.93E-04 | 1.88E-05 | 4.00E-03 |
|  | Rhizophydiales | 1.50E-03 | 2.96E-04 | 1.20E-02 |
| genus | *Hyaloraphidium* | 4.44E-03 | 1.07E-02 | 9.99E-04 |
|  | *Papiliotrema* | 1.38E-04 | 0.00E+00 | 9.99E-04 |
|  | *Penicillium* | 2.66E-04 | 0.00E+00 | 9.99E-04 |
|  | *Rhodotorula* | 2.51E-04 | 0.00E+00 | 9.99E-04 |
|  | *Smittium* | 6.93E-04 | 1.88E-05 | 6.99E-03 |
|  | *Saccharomyces* | 4.18E-05 | 0.00E+00 | 1.56E-02 |
| s4 vs s6 |  | Mean(s4) | Mean(s6) | P value |
| phylum | Chytridiomycota | 7.68E-01 | 4.16E-01 | 9.99E-04 |
|  | Kickxellomycota | 6.93E-04 | 2.27E-05 | 2.00E-03 |
|  | Rozellomycota | 6.29E-04 | 1.02E-03 | 2.20E-02 |
| class | Chytridiomycetes | 6.69E-01 | 3.39E-01 | 9.99E-04 |
|  | Monoblepharidomycetes | 4.44E-03 | 2.68E-02 | 9.99E-04 |
|  | Sordariomycetes | 2.29E-04 | 1.14E-03 | 9.99E-04 |
|  | Harpellomycetes | 6.93E-04 | 2.27E-05 | 3.00E-03 |
|  | Rozellomycotina_cls_Incertae_sedis | 5.32E-04 | 9.63E-04 | 6.99E-03 |
| family | Trichosphaeriaceae | 1.54E-04 | 9.94E-04 | 9.99E-04 |
|  | Legeriomycetaceae | 6.93E-04 | 2.27E-05 | 3.00E-03 |
|  | Saccharomycetaceae | 4.18E-05 | 0.00E+00 | 6.24E-03 |
| genus | *Hyaloraphidium* | 4.44E-03 | 2.68E-02 | 9.99E-04 |
|  | *Nigrospora* | 1.54E-04 | 9.94E-04 | 9.99E-04 |
|  | *Penicillium* | 2.66E-04 | 0.00E+00 | 9.99E-04 |
|  | *Smittium* | 6.93E-04 | 2.27E-05 | 3.00E-03 |
|  | *Saccharomyces* | 4.18E-05 | 0.00E+00 | 6.24E-03 |
| species | *Candida_railenensis* | 6.45E-04 | 0.00E+00 | 9.99E-04 |
|  | *Hyaloraphidium_curvatum* | 4.44E-03 | 2.68E-02 | 9.99E-04 |
|  | *Nigrospora_zimmermanii* | 1.54E-04 | 9.94E-04 | 9.99E-04 |
|  | *Penicillium_lemhiflumine* | 2.66E-04 | 0.00E+00 | 9.99E-04 |
|  | *Smittium_morbosum* | 6.93E-04 | 2.27E-05 | 9.99E-04 |
|  | *Saccharomyces_mikatae* | 4.18E-05 | 0.00E+00 | 6.24E-03 |
|  | *Cladosporium_endophytica* | 1.40E-04 | 1.37E-05 | 3.00E-02 |
| s5 vs s6 |  | Mean(s5) | Mean(s6) | P value |
| phylum | Ascomycota | 1.45E-03 | 3.77E-03 | 9.99E-04 |
|  | Chytridiomycota | 6.20E-01 | 4.16E-01 | 9.99E-04 |
| family | Aspergillaceae | 0.00E+00 | 1.34E-04 | 9.99E-04 |
|  | Cladosporiaceae | 6.45E-04 | 1.55E-03 | 9.99E-04 |
|  | Cordycipitaceae | 5.38E-06 | 1.45E-04 | 9.99E-04 |
|  | Trichosphaeriaceae | 4.14E-04 | 9.94E-04 | 8.99E-03 |
|  | Rhynchogastremataceae | 0.00E+00 | 3.71E-05 | 1.61E-02 |
|  | Ustilaginaceae | 2.88E-05 | 2.14E-04 | 3.60E-02 |
| genus | *Aspergillus* | 0.00E+00 | 1.34E-04 | 9.99E-04 |
|  | *Cladosporium* | 6.45E-04 | 1.55E-03 | 9.99E-04 |
|  | *Hyaloraphidium* | 1.07E-02 | 2.68E-02 | 9.99E-04 |
|  | *Nigrospora* | 4.14E-04 | 9.94E-04 | 9.99E-03 |
|  | *Papiliotrema* | 0.00E+00 | 3.71E-05 | 1.61E-02 |
|  | *Moesziomyces* | 2.88E-05 | 2.14E-04 | 4.00E-02 |
